# Supplementary material for: Content-rich biological network constructed by mining PubMed abstracts
Source: BMC Bioinformatics. 2004 Oct 8;5:147. doi: 10.1186/1471-2105-5-147 (PMC528731; doi:10.1186/1471-2105-5-147)
Supplement: Additional File 2 — The original results of the above study (non-essential files are deleted to keep the file size under the limit set by BMC bioinformatics). [file 1471-2105-5-147-S2.bz2 › chilibotAdditionalFile2/dip05/585ID9311977E10953/html/RNH_ANG.html]

 


 **RNH** and **ANG** 
  
Found 69 abstracts in PubMed, retrieved 05.  
 

 What does Google say? 
 PDF only 
| .edu only 

---

**Interactive relationship** (e.g. stimulation, inhibition, etc)

**Neutral relationship**- Genetic screen to dissect protein protein interactions ribonuclease inhibitor  [ **RNH** ]  ribonuclease A  [ **ANG** ]  as a model system.  Ref: 12431438 Methods, 2002

**Non-interactive relationship** (e.g. studied together, co-existance, homology, etc.)

- The efficacy of this approach is demonstrated with the ribonuclease inhibitor  [ **RNH** ]  protein and ribonuclease A  [ **ANG** ] , which form a complex with an equilibrium dissociation constant in the femtomolar range.  Ref: 12431438 Methods, 2002
